# Detection of Selection Signatures on the X Chromosome in Three Sheep Breeds

**DOI:** 10.3390/ijms160920360

**Published:** 2015-08-28

**Authors:** Caiye Zhu, Hongying Fan, Zehu Yuan, Shijin Hu, Li Zhang, Caihong Wei, Qin Zhang, Fuping Zhao, Lixin Du

**Affiliations:** 1 National Center for Molecular Genetics and Breeding of Animal, Institute of Animal Sciences, Chinese Academy of Agricultural Sciences, No. 2 Yuanmingyuan West Rd., Haidian, Beijing 100193, China; E-Mails: zhucaiye2015@sina.com (C.Z.); fan_hying@163.com (H.F.); yuanzehu_caas@163.com (Z.Y.); hushijinde@126.com (S.H.); zhangli712@263.net (L.Z.); xiaozhu1211@eyou.com (C.W.); 2 College of Animal Science and Technology, China Agricultural University, Beijing 100193, China; E-Mail: qzhang@cau.edu.cn; 3 College of Animal Science and Technology, Gansu Agricultural University, Lanzhou 730070, China

**Keywords:** selection signature, X chromosome, sheep

## Abstract

Artificial selection has played a critical role in animal breeding. Detection of artificial selection footprints in genomic regions can provide insights for understanding the function of specific phenotypic traits and better guide animal breeding. To more fully understand the relationship between genomic composition and phenotypic diversity arising from breed development, a genome-wide scan was conducted using an OvineSNP50 BeadChip and integrated haplotype score and fixation index analyses to detect selection signatures on the X chromosome in three sheep breeds. We identified 49, 34, and 55 candidate selection regions with lengths of 27.49, 16.47, and 25.42 Mb in German Mutton, Dorper, and Sunit sheep, respectively. Bioinformatics analysis showed that some of the genes in these regions with selection signatures, such as *BMP15*, were relevant to reproduction. We also identified some selection regions harboring genes that had human orthologs, including *BKT*, *CENPI*, *GUCY2F*, *MSN*, *PCDH11X*, *PLP1*, *VSIG4*, *PAK3*, *WAS*, *PCDH19*, *PDHA1*, and *SRPX2*. The *VSIG4* and *PCDH11X* genes are associated with the immune system and disease, *PDHA1* is associated with biosynthetic related pathways, and *PCDH19* is expressed in the nervous system and skin. These genes may be useful as candidate genes for molecular breeding.

## 1. Introduction

Artificial selection has played a significant role in the domestication of livestock. Approximately 11,000 years ago, domesticated animals began to appear in the Fertile Crescent [1]. Sheep, the first livestock known to be domesticated, were initially reared for their meat before their breeding became specialized for secondary products, such as wool, which occurred between 4000 and 5000 years ago [2]. Domestication reshaped the behavior, morphology, and genetics of the involved livestock. For example, two studies found that artificial selection changed sheep coat pigmentation, horn morphology and growth developmental traits [3,4] using a genome scan of recent positive selection signatures in three sheep populations.

Selection has many effects on the genome. Positive selection can improve advantageous allele frequencies and fix them within a population [5]. As a result, polymorphism at a selection site is reduced in the population. With the development of high-density single nucleotide polymorphism (SNP) chips and high-throughput genotyping technology, a number of statistics have been used to explore selection signatures on genes and the genome [6]. For instance, signatures indicating diversified selection among breeds are found in genomic regions associated with traits related to the standard criteria for breeds, such as coat color and ear morphology. Other selection signals are found in genomic regions such as quantitative trait loci and genes associated with production traits, including reproduction, growth, and fat deposition. Some selection signatures in sheep are associated with regions showing evidence of introgression from Asian breeds. When European sheep breeds were compared with the wild boar, genomic regions with high levels of differentiation were found to harbor genes related to bone formation, growth, and fat deposition [7].

Selection signatures can be detected through the variation of allele frequency and decay of linkage disequilibrium. To date, some related methods have been put forward and can be classified into categories of linkage disequilibrium and site-frequency spectrum [8]. The integrated haplotype score (iHS) [9] and F-statistics (F_ST_) [10] are extensively used in identifying selection signatures. The iHS has been used mainly to reveal the selection signatures (within-population methods) using information from a single population, whereas F_ST_ has been used primarily to detect selection signatures between populations. The iHS is based on linkage disequilibrium theory and can detect regions with a rapidly increased frequency of the derived allele at selected sites [9]. The F_ST_, first used to evaluate population variation through the DNA polymorphism of a population, is currently based on a Bayesian hierarchical model [11]. McRae *et al*. [12] used F_ST_ to identify 14 novel regions associated with resistance or susceptibility to gastrointestinal nematodes in sheep.

The X chromosome has a high density of genes and thus may be a good target for detecting selection signatures. Rubin *et al*. [13] pointed out that the X chromosome should be solely analyzed for the identification of selection signatures, and only sows should be used because sex chromosomes and autosomes, even between genders, are subjected to different selective pressures and have different effective population sizes. The X chromosome undergoes more drift than autosomes, as its effective population size is three-quarters that of autosomes [14]. The X chromosome is more specialized than an autosome and plays an important role in evolution of human and animals. Studies have shown that selection on the X chromosome reduces the genomic diversity to a greater extent than that on autosomes (19%–26%) [15]. Ma *et al*. [6] studied selection footprints on the X chromosome in pigs and determined that genes relevant to meat quality, reproduction, and the immune system were found in potential selection regions. Moradi *et al*. [16] used the OvineSNP50 BeadChip to scan selective sweeps in thin and fat tail sheep and found increased homozygosity in selection regions in favor of fat tail breeds on chromosomes 5 and X. Taking into account the sex-specific dosage compensation, the selection pressure on the X chromosome is higher than that on the autosomes, indicating that genes on the X chromosome are under more direct and effective selection [17,18]. The X chromosome in sheep contains several genes relevant to desirable breeding traits, including those related to tail fat deposition [19], further indicating that this is a good target chromosome for examining selection signatures in sheep.

Therefore, in the present study, within-population (iHS) and between-population (F_ST_) methods were used to search the whole X chromosome in three breeds of sheep for signatures of positive selection using the OvineSNP50 BeadChip array, followed by candidate gene enrichment analysis and gene annotation to elucidate the biological functions of the selection signature.

## 2. Results

### 2.1. Markers and Core Haplotypes

**Figure 1:**
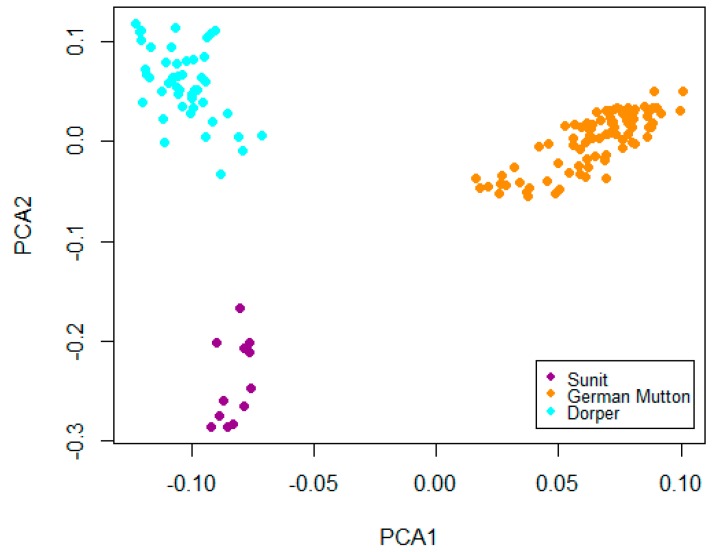
Animal clusters based on principal component analysis (PCA) using individuals. Plots for the first (PCA1) and second (PCA2) components reveal the clustering of 148 animals from German Mutton, Dorper and Sunit.

After quality control and PCA analyses (Figure 1), 89 German Mutton, 47 Dorper, and 12 Sunit ewes were used in the final analyses. A total of 1226 SNPs were obtained per breed. The average distance between two SNPs was 110.11 kb.

### 2.2. Empirical Distribution of Test Statistics

**Figure 2:**
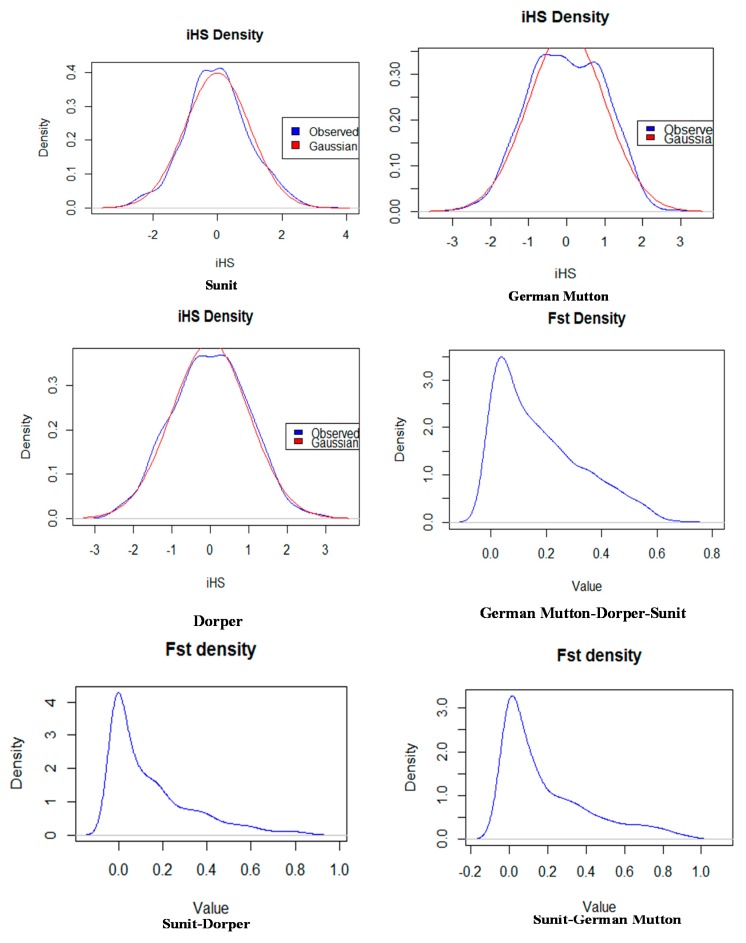

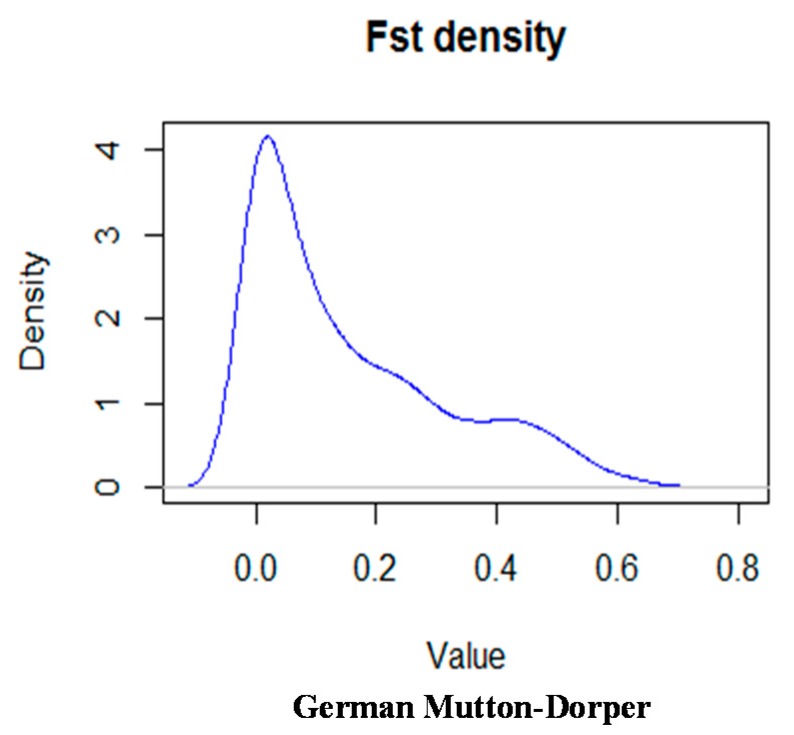
Empirical distribution of test statistics within the three individual populations (iHS) and between (F_ST_) the German Mutton, Dorper and Sunit ewe populations.

The empirical distributions of the two test statistics for each breed and breed pair were clearly observed. Figure 2 shows the distributions of the iHS and F_ST_ values on the X chromosome of German Mutton sheep and pairwise for German Mutton, Dorper, or Sunit. Table S1, Table S2, Table S3 and Table S4 show the true values for each SNP in every breed. The standardized iHS and F_ST_ values approximately followed a standard normal distribution, as pointed out by Sabeti *et al*. [20]. Moreover, the distributions of the iHS test statistics indicated that the other individual breeds and the comparison of breed pairs with one another showed similar results.

### 2.3. Identification of Recent Selection Signatures on the X Chromosome

**Figure 3:**
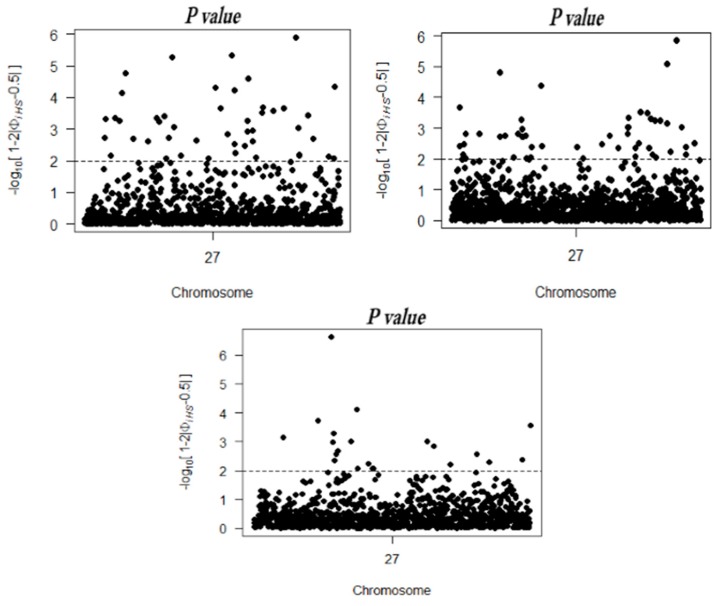
The X chromosome distribution of *P_iHS_* values in the three breeds. The dotted line was denoted as the threshold, indicating significant regions.

The scanning of the X chromosome for selection signatures in the three sheep breeds was conducted using between- and within-population methods. First, the within-population method iHS was used to look for selection signatures in the three breeds. The iHS scores were computed at each SNP over the whole genome using haplotypes. Figure 3 depicts the distribution of the *P_iHS_* value on the X chromosome to visualize the distribution of the selection signatures. As shown in Table 1, there were 51, 21, and 46 outliers identified in German Mutton, Dorper, and Sunit, respectively.

**Table 1:** Summary of selection signatures detected using iHS and F_ST_ in three sheep breeds.

Items	iHS			F_ST_			
	G M	D	S	G M-D-S	G M-D	S-G M	S-D
Number of SNPs	51	21	46	12	12	12	12
Number of regions	35	17	37	10	9	11	9
Average length (Mb)	0.43	0.44	0.43	0.43	0.43	0.40	0.45
Total length (Mb)	14.78	7.59	15.93	4.35	3.89	4.44	40.3

(G M represents German Mutton; D represents Dorper; S represents Sunit).

**Figure 4:**
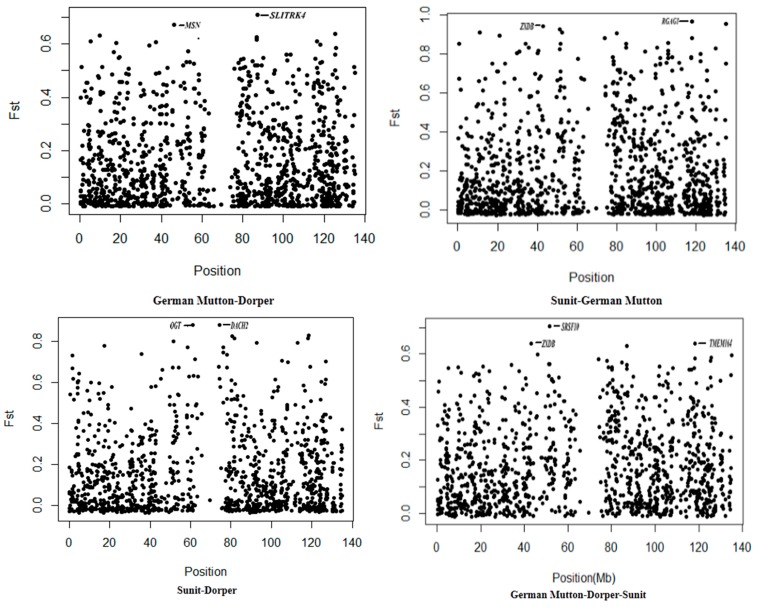
The X chromosome distribution of F_ST_ values.

For the F_ST_ test, the population genetic differentiation at each genetic marker was detected. Because the F_ST_ empirical distribution of a single site was similar to a chi-squared (*χ^2^*) distribution with two or three degrees of freedom, the boxplot method was used to identify outliers in the genome-wide selection signal. Figure 4 depicts the distribution of the F_ST_ values on the X chromosome. A total of 12 outlier sites was detected in the three breeds.

### 2.4. Candidate Selection Regions

A region on the X chromosome having a false discovery rate (FDR) less than 0.1 for both methods, or an FDR less than 0.05 for one method, was considered a candidate selection region. As shown in Table 1, using the iHS test, 35, 17, and 37 candidate selection regions with lengths of 14.78, 7.59, and 15.93 Mb were identified in German Mutton, Dorper, and Sunit, respectively. As shown in Table 1, using the F_ST_ test, 10 candidate selection regions with lengths of 4.35 Mb were identified for German Mutton-Dorper-Sunit. Overall, approximately 19.33, 11.82, and 20.10 Mb selection regions were detected in German Mutton, Dorper, and Sunit. The Sunit, a representative Chinese indigenous breed, shared approximately 5.46 Mb candidate selection regions with German Mutton. This was less than the overlapping regions between Sunit and Dorper. In addition, there were approximately 7.29 Mb candidate selection regions shared by German Mutton and Dorper, with an overlapping length longer than that shared by Sunit and German Mutton or by Sunit and Dorper.

### 2.5. Identification of Candidate Genes in Selection Regions and Functional Analysis

The genes in the selection regions were identified using the Ovis_aries_3.1 database. After a comparison analysis, 102, 76, 102 genes were detected within regions positive for selection signatures in German Mutton, Dorper, and Sunit, respectively. Table 2 summarizes the genes in regions with the top five significant |*iHS*| values on the X chromosomes of the three breeds.

**Table 2:** Selection regions on the X chromosome and corresponding genes for the top five significant iHS values in each sheep breed.

Breed	Region (Mb)	|*iHS*|	Gene
German Mutton	122.16–122.56	5.85	*TCEAL4*, *TCEAL1*, *MORF4L2*, *GLRA4*, *PLP1*, *RAB9B*, *TMSB15B*, *CYSLTR1*
	118.98–119.38	5.07	*NXT2*
	19.83–20.23	4.80	*-*
	40.51–40.91	4.39	*FAM192A*
	3.77–4.17	3.66	*-*
Dorper	30.58–30.98	6.63	*LAMP1*
	41.36–41.76	4.12	*EFHC2*
	23.67–24.07	3.72	*-*
	134.90–135.30	3.56	*PABPC5*
	31.27–31.67	3.29	*-*
Sunit	103.45–103.85	3.63	*TEX13A*
	31.98–32.38	3.44	*TMEM47*
	40.13–40.53	3.32	-
	116.80–117.2	3.30	*TRPC5*, *ALG13*
	94.82–95.22	3.25	-

The locus with the greatest F_ST_ value (0.705) was within the DNA region of the *SRSF10* gene. The genes that overlapped with three other F_ST_ peaks were *TMEM164*, *ZXDB*, and *SLITRK2*. Twelve other SNPs with significant F_ST_ values also existed within selection regions. The genes harbored in the selection regions are shown in Table 3.

**Table 3:** Selection and candidate genes detected by F_ST_ among the three breeds.

Breed Pair	Position (Mb)	F_ST_ Value	Gene Name
German Mutton-Dorper-Sunit	51.51–51.91	0.71	*SRSF10*, *DGKK*, *CCNB3*
	117.91–118.31	0.64	*TMEM164*
	42.80–43.21	0.64	*ZXDB*
	86.91–87.31	0.63	*SLITRK2*
	45.82–46.22	0.59	*MSN*, *MMD*, *VSIG4*, *HEPH*
	134.78–135.18	0.59	*PCDH11X*, *ZGC*:*112234*, *PABPC5*
	125.43–125.83	0.58	*TIMM8A*, *BTK*, *RPL36A*, *GLA*, *HNRNPH2*, *ARMCX4*, *CSNK1A1*, *ARMCX1*, *ARMCX6*, *ARMCX3*, *ARMCX2*
	117.84–118.24	0.58	*RGAG1*, *AMMECR1*
	73.69–74.09	0.57	*CHM*, *DACH2*
	77.77–78.17	0.57	*FAM58A*, *ATP2B3*, *BGN*, *HAUS7*, *TREX2*, *ZNF275*, *KIR3DL1*
	125.15–125.55	0.57	*XKRX*, *ARL13A*, *TRMT2B*, *TMEM35*, *CENPI*, *DRP2*, *TAF7L*
	84.41–84.81	0.56	*SLITRK2*, *SLITRK4*
German Mutton-Dorper	86.65–87.30	0.71	*SLITRK4*
	45.82–46.22	0.67	*MSN*, *MMD*, *VSIG4*, *HEPH*
	125.15–125.55	0.64	*XKRX*, *ARL13A*, *TRMT2B*, *TMEM35*, *CENPI*, *DRP2*, *TAF7L*
	9.35–9.75	0.63	*-*
	4.85–5.25	0.61	*PNPLA4*
	116.23–116.63	0.61	*-*
	37.26–37.66	0.61	*BCOR*, *DUT*
	17.56–17.96	0.60	*RPS6KA3*
	117.84–118.24	0.59	*RGAG1*, *AMMECR1*
Sunit-German Mutton	117.91–118.31	0.97	*RGAG1*, *AMMECR1*, *TMEM164*
	134.78–135.18	0.95	*PCDH11X*, *ZGC*:*112234*, *PABPC5*
	42.80–43.20	0.94	*ZXDB*
	51.17–51.61	0.93	*SHROOM4*, *SRSF10*
	10.63–11.03	0.91	*EGFL6*, *RAD51L3*
	52.36–52.76	0.91	*USP27X*, *SUMO2*, *PPP1R3F*, *FOXP3*, *CCDCC22*, *CACNA1F*, *SYP*, *PRICKLE3*, *PLP2*, *MAGIX*, *GPKOW*, *RPL36*, *WDR45*, *PRAF2*
	79.89–80.29	0.90	*CD99L2*, *MTMR1*, *MTM1*, *MAMLD1*
	20.82–21.22	0.89	*PRDX4*, *ACOT9*, *SAT1*
	73.69–74.09	0.88	*CHM*, *DACH2*
	117.91–118.31	0.88	*RGAG1*, *AMMECR1*, *TMEM164*
Sunit-Dorper	60.80–61.20	0.87	*OGT*, *ACRC*, *CXCR3*, *DMRTC2*
	74.29–74.69	0.87	*DACH2*
	118.09–118.49	0.82	*AMMECR1, TMEM164*
	80.30–80.76	0.82	*MAMLD1*
	117.72–118.12	0.81	*CHRDL1*, *RGAG1*
	81.49–81.89	0.81	*AFF2*
	51.51–51.91	0.80	*SRSF10*, *DGKK*, *CCNB3*
	112.66–113.06	0.79	*PLS3*
	92.61–93.01	0.79	*-*
	17.08–17.48	0.77	*MAP3K15*, *RPL17*, *SH3KBP1*, *CXORF23*

DAVID v2.1 was used to conduct the GO and KEGG pathway enrichment analyses to investigate the functions of the candidate genes. After this enrichment analysis, followed by the Benjamini correction procedure, there were almost no significant functional terms.

The genes were found to be involved in metabolism, muscle development, and reproduction based on information in the NCBI gene database, although some genes were not entirely harbored within the potential selection regions. Among them, *BMP15*, identified by iHS, overlapped with the potential selection region of 51.07–51.91 Mb and functions in reproduction [21].

## 3. Discussion

In recent years, extensive research using SNP chip array data to detect positive selection signatures has made considerable progress [9,22,23,24,25]. During this same time, a number of statistical methods have been developed to identify selection regions in the genome. To increase the accuracy of detection, we used two methods in the present study to detect selection regions on the X chromosome: iHS and F_ST_ approaches. The iHS relies on the EHH statistic, providing a more powerful approach to identify selection footprints at loci that are fixed or probably fixed [26]. The iHS test is based on linkage disequilibrium and is dependent on SNP spacing and frequency because it is a multi-marker test [16]. On the X chromosome, 1226 SNPs may not affect the accuracy of the iHS. Additionally, the power of the iHS method also relies on ancestral allele information, which is available for only a portion of the SNPs on the ovine chip [16]. However, Zhao *et al*. [27] used iHS and F_ST_ to successfully identify selection signatures in dairy and beef cattle .The F_ST_ measures population differentiation by detecting allele frequencies at a locus using a between-population method [28]. Detecting recent positive selection with F_ST_ is complicated because the distribution of the genetic variation due to selection can be difficult to distinguish from that which arises after certain demographic events [15]. However, ascertainment bias and demographic events would be expected to change patterns of F_ST_ in the same way genome-wide, whereas F_ST_ values only in selected and nearby loci would be altered by selection events [29,30,31,32].

In this study, the results obtained using the iHS method followed strict distributions. By contrast, the results obtained using the F_ST_ approach did not follow strict distributions, but followed an approximately normal distribution. This difference indicates that the risk of false positives when using the traditional significance test remains high because of the uncertainty in the null distribution for the test statistic [6]. For this reason, a boxplot strategy was used to determine the upper and lower threshold values, confirming the outliers for the F_ST_ values at each SNP locus. Thus, the results of the methods used to detect selection signatures on the X chromosome were subjected to strict criteria to prevent the occurrence of false positives. As stated, for the F_ST_ statistic, the boxplot strategy [29] was adopted to define upper and lower threshold F_ST_ values for each SNP locus to determine outliers. We first calculated the distribution of the X chromosome F_ST_ interquartile range; then, F_ST_ values greater than the upper threshold or less than the lower threshold values were defined as outliers.

In this study, 49, 34, and 55 selection regions were detected in German Mutton, Dorper, and Sunit breeds, respectively. German Mutton sheep were imported into China from Germany at the end of 20th century, and Dorper sheep from Australia in 2001 [30]. As these livestock populations migrated across the globe, they encountered numerous environments, each with unique ecological conditions. The populations were exposed to artificial selection through breeding programs. Hence, their genomes would be expected to be marked with many signals of positive selection. Sunit sheep are indigenous to northern China and are used for both their meat and fat in the Inner Mongolia Autonomous Region of China. Sunit sheep have adapted to the natural conditions of the Gobi Desert after approximately 800 years of natural and artificial selection. German Mutton shared approximately 7.29 Mb candidate selection regions with Dorper. This is a longer overlapping length than that shared by Sunit and German Mutton, which shared 5.46 Mb, or Dorper and Sunit, which shared approximately 5.90 Mb selection regions. This result suggests that selection intensity may be greater for German Mutton and Dorper than for Sunit sheep.

Following the Benjamini correction for the enrichment analysis of the genes located in candidate selection regions, no significant functional terms were detected. However, some GO terms with *p* values less than 0.05 were related to biological process, molecular functions, and cellular components, indicating that some traits may have undergone selection during the domestication of these sheep. We also detected some genes associated with the immune system on the X chromosome in sheep, consistent with the identification of selection footprints on the X chromosome in pigs [6].

Among our candidate genes, *VSIG4* was identified and characterized by Helmy *et al*. [31] to be a complement receptor in the immunoglobulin superfamily, binding complement fragments C3b and iC3b to remove pathogenic microorganisms. The *PCDH19* gene was another candidate gene for selection detected in our study, and it is expressed in the nervous system and skin and its derivative tissues [32]. The *BTK* gene is known to function in adaptive immunity, mainly in B cell signaling pathways, playing a key role in B cell proliferation, development, differentiation, survival, and apoptosis [33,34]. Demars *et al*. [35] identified two novel *BMP15* mutations responsible for an atypical hyperprolificacy phenotype in sheep.

To date, a number of studies have identified selection signatures in sheep [36,37], but these studies focused on autosomal genes. Amaral *et al*. [38] used sequencing of pooled DNA to detect selection signatures genome-wide, but found it was difficult to analyze the isolated X chromosome. Rubin *et al*. [13] proposed that the X chromosome should be separately analyzed to detect selection signatures, as we did in this study. Thus, using iHS and F_ST_, we detected 222 genes on the X chromosome in sheep with signatures indicating that they had undergone selection during domestication. These genes can be used as molecular markers in future sheep breeding.

## 4. Experimental Section

### 4.1. Experimental Animals and DNA Samples

The sheep population initially consisted of 161 (71 males and 90 females) German Mutton, 99 (49 males and 50 females) Dorper, and 69 (57 males and 12 females) Sunit. After a principal component analysis (PCA) was performed to identify population structure and the relatedness of animals, 148 females, including 89 German Mutton, 47 Dorper, and 12 Sunit, were finally chosen for selection signature identification on the X chromosome.

Blood samples were collected from six-month-old lambs using standard methods. Whole genomic DNA was extracted from blood samples using a TIANamp Blood DNA kit (Tiangen Biotech Co., Ltd., Beijing, China).

### 4.2. Genotyping and Quality Control

Genomic DNA was genotyped using the Illumina OvineSNP50 BeadChip containing 54,241 SNPs with an average gap spacing distance of 50.9 kb. The genotyping platform used was the Infinium II Multi-Sample Assay (Illumina, Inc., San Diego, CA, USA). The SNP chips were scanned using iScan, and the data were analyzed using GenomeStudio software (Illumina).

PLINK software (v1.07; http://pngu.mgh.harvard.edu/purcell/plink) was used to control the quality of the X chromosome genotype data. An individual was removed if the call rate was less than 90% or the sample was a duplicate. A SNP locus was excluded if (1) the SNP call rate was less than 90%; (2) its minor allele frequency was less than 0.05; or (3) it did not obey Hardy-Weinberg equilibrium (*p* value < 10^−6^). After quality control, BEAGLE software [39] was used to impute the missing genotypes and infer haplotypes.

### 4.3. Analyses Integrated Haplotype Score

The iHS, calculated as described by Voight *et al*. [9], was defined as the log of the ratio of the integrated extended haplotype homozygosity (EHH) score for haplotypes centering the ancestral allele to the integrated EHH score for haplotypes centering the derived allele. The iHS score was computed at X chromosome SNPs for each breed using the R package “rehh” [40]. The formula for the standardized iHS is as follows:

(1) $$iHS=\frac{\ln\left(\frac{iHH_{A}}{iHH_{D}}\right)\text{-}E\left[\ln\left(\frac{iHH_{A}}{iHH_{D}}\right)\right]}{SD\left[\ln\left(\frac{iHH_{A}}{iHH_{D}}\right)\right]}$$

where iHHA and iHHD represent the integrated EHH score for ancestral and derived core alleles, respectively.

### 4.4. Population Differentiation Index

The classic measure of population genetic differentiation, F_ST_, was used to detect signatures of diversifying selection, based on genetic polymorphism data. Here, F_ST_ was calculated as described by MacEachern *et al*. [41]:

(2) $$F_{\text{ST}}=\frac{H_{\text{T}}-H_{\text{S}}}{H_{\text{T}}}$$

where *H*_T_ represents the expected heterozygosity for the overall total population such that

(3) $$H_{\text{T}}=1-\sum \left(p^{2}-q^{2}\right)$$

where *p* and *q* denote the frequency of alleles A1 and A2 over the total population.

In Equation (2), *H*_S_ represents the expected heterozygosities in subpopulations and is calculated as follows:

(4) $$H_{\text{S}}=\frac{\sum_{i=1}^{n}H_{\exp_{i}}\times n_{i}}{N_{\text{Total}}}$$

with *H*_exp*i*_ denoting expected heterozygosity and *n_i_* denoting the sample size in subpopulation *i*.

### 4.5. Identifying the Region in the X Chromosome under Selection

For F_ST_, A boxplot strategy was used to determine the upper and lower threshold values to confirm outliers of the F_ST_ values for each SNP locus.

First, the interquartile range (Q) of the F_ST_ empirical distribution on X chromosome was calculated as

(5) $$Q=F^{U}+F^{L}$$

with *F^U^* and *F^L^* representing the upper and lower interquartile ranges, respectively. The upper (UL) and lower (LL) threshold values were then calculated as follows:

(6) $$UL=F^{U}+1.5Q$$

(7) $$LL=F^{L}-1.5Q$$

All values greater than the upper threshold or less than the lower threshold values were defined as outliers.

For iHS, the thresholds of empirical cutoffs for the X chromosome were based on the autosomal cutoffs. The threshold of |*iHS*| on the X chromosome was |*iHS*| > 2.

### 4.6. Enrichment Analysis

Kyoto Encyclopedia of Genes and Genomes (KEGG) pathway mapping, molecular function, cellular components, and biological process were determined for the candidate selection regions. An abundant database of human genomic information was referred to identify genes on the sheep genome using the many available annotations on the sheep genome. The program Database for Annotation, Visualization and Integrated Discovery (DAVID) 6.7 (http://david.abcc.ncifcrf.gov/) [42] was used to generate the homology gene set and gene enrichment analysis.

### 4.7. Gene Annotation

In the selection region, the outlier or selection footprint was extended approximately 100 kb in the upstream and downstream directions. The National Center for Biotechnology Information (http://www.ncbi.nlm.nih.gov/gene/) database and the most recent sheep genome Ovis_aries_v3.1 [43] (http://www.livestockgenomics.csiro.au/sheep/oar3.1.php) were used to identify the biological function of genes in the selection regions. In addition, the genomic information of other species, including human, mouse, and bovine, were used to predict gene function.

## 5. Conclusions

In this study, we were able to identify many novel regions and genes on X chromosome by different methods, we demonstrated that X chromosome has undergo selection in the process of sheep domesticated.

## Supplementary Materials

Supplementary materials can be found at http://www.mdpi.com/1422-0067/16/09/20360/s1.
